# Altered microRNA expression in COVID-19 patients enables identification of SARS-CoV-2 infection

**DOI:** 10.1371/journal.ppat.1009759

**Published:** 2021-07-28

**Authors:** Ryan J. Farr, Christina L. Rootes, Louise C. Rowntree, Thi H. O. Nguyen, Luca Hensen, Lukasz Kedzierski, Allen C. Cheng, Katherine Kedzierska, Gough G. Au, Glenn A. Marsh, Seshadri S. Vasan, Chwan Hong Foo, Christopher Cowled, Cameron R. Stewart

**Affiliations:** 1 CSIRO Health & Biosecurity, Australian Centre for Disease Preparedness, Geelong, Victoria, Australia; 2 Department of Microbiology and Immunology, University of Melbourne, at the Peter Doherty Institute for Infection and Immunity, Melbourne, Victoria, Australia; 3 Faculty of Veterinary and Agricultural Sciences, University of Melbourne, Melbourne, Victoria, Australia; 4 School of Public Health and Preventive Medicine, Monash University, Melbourne, Victoria, Australia; 5 Infection Prevention and Healthcare Epidemiology Unit, Alfred Health, Melbourne, Victoria, Australia; 6 Global Station for Zoonosis Control, Global Institution for Collaborative Research and Education (GI-CoRE), Hokkaido University, Sapporo, Japan; 7 Department of Health Sciences, University of York, York, United Kingdom; 8 Exios Bio LLC, Conshohocken, Pennsylvania, United States of America; Erasmus Medical Center, NETHERLANDS

## Abstract

The host response to SARS-CoV-2 infection provide insights into both viral pathogenesis and patient management. The host-encoded microRNA (miRNA) response to SARS-CoV-2 infection, however, remains poorly defined. Here we profiled circulating miRNAs from ten COVID-19 patients sampled longitudinally and ten age and gender matched healthy donors. We observed 55 miRNAs that were altered in COVID-19 patients during early-stage disease, with the inflammatory miR-31-5p the most strongly upregulated. Supervised machine learning analysis revealed that a three-miRNA signature (miR-423-5p, miR-23a-3p and miR-195-5p) independently classified COVID-19 cases with an accuracy of 99.9%. In a ferret COVID-19 model, the three-miRNA signature again detected SARS-CoV-2 infection with 99.7% accuracy, and distinguished SARS-CoV-2 infection from influenza A (H1N1) infection and healthy controls with 95% accuracy. Distinct miRNA profiles were also observed in COVID-19 patients requiring oxygenation. This study demonstrates that SARS-CoV-2 infection induces a robust host miRNA response that could improve COVID-19 detection and patient management.

## Author summary

While it is recognized that the host response to infection plays a critical role in determining the severity and outcome of COVID-19, the host microRNA (miRNA) response to SARS-CoV-2 infection is poorly defined. Here we have used next-generation sequencing and bioinformatics to profile circulating miRNAs in 10 COVID-19 patients that were sampled longitudinally over time. COVID-19 was associated with altered expression of 55 plasma miRNAs, with miR-776-3p and miR-1275 among the most strongly down-regulated, and miR-4742-3p, miR-31-5p and miR-3215-3p the most up-regulated. An artificial intelligence methodology was used to identify a miRNA signature, consisting of miR423-5p, miR-23a-3p, miR-195-5p, which could independently classify COVID-19 patients from healthy controls with 99.9% accuracy. When applied to the ferret model of COVID-19, the same signature classified COVID-19 cases with 99.8% accuracy and could distinguish between COVID-19 and influenza A(H1N1) infection with >95% accuracy. In summary this study profiles the host miRNA response to COVID-19 and suggests that the measurement of select host molecules may have potential to independently detect disease cases.

## Main

As of February 2021, the COVID-19 pandemic, caused by infection with severe acute respiratory syndrome-associated coronavirus-2 (SARS-CoV-2) has resulted in over 107 million cases and 2.35 million deaths worldwide [1]. The outcome of SARS-CoV-2 infection varies widely from asymptomatic to severe disease associated with acute respiratory distress syndrome (ARDS) and death [2]. Several studies have established that host responses to infection play a critical role in determining disease outcomes in infected patients. For example, hyper-inflammatory responses including high levels of circulating cytokines and chemokines (particularly interleukin (IL)-6, IL-8, and tumor necrosis factor (TNF)-α), lymphopenia and immune cell infiltration in infected organs are considered major determinants of COVID-19 severity [3–6]. As there are currently no approved curative treatments for COVID-19, the characterisation of host factors associated with SARS-CoV-2 pathogenesis is critically important for the design of novel therapies.

MicroRNAs (miRNAs) are a class of non-coding RNAs that regulate endogenous gene expression at the post-transcriptional level. In most instances, miRNAs function by interacting with the 3′ untranslated region (3′ UTR) of target mRNAs to induce degradation and translational repression [7]. There are currently over 2,600 human miRNAs listed in the miRNA registry (miRBase, version 22) [8] which are estimated to collectively regulate 60% of all human protein-coding genes [9]. The scientific rationale for investigating miRNAs during viral infections is two-fold. Firstly, miRNA profiles offer unique insight into cellular pathways associated with virus replication and pathogenesis. For instance, the human coronavirus OC43 potentiates NF-kB activation during infection by binding and sequestering miR-9, a negative regulator of NF-kB [10]. There is also evidence that coronaviruses co-opt the host miRNAs response to subvert antiviral immune responses. Infection by the *Alphacoronavirus* transmissible gastroenteritis virus (TGEV) downregulates miR-30a-5p expression, which disrupts the type I interferon response against TGEV [11]. Secondly, the characterisation of host miRNAs responses to virus infection informs the development of biomarkers for improved disease detection and forecasting of disease outcome [12]. Several pathogenic viruses, including SARS-CoV-1, induce changes to the circulating host miRNA profile [13–17]. Interestingly, host miRNA responses to SARS-CoV-1 and influenza A virus differ based on virus type and pathogenicity [13], highlighting the potential for miRNAs to serve as diagnostic or prognostic biomarkers.

In this study we have investigated the circulating miRNA profiles in the plasma of ten COVID-19 patients and ten age and gender matched healthy donors. We observed that among patient samples collected during early-stage disease, COVID-19 induced differential expression of 55 host-encoded microRNAs, with miR-31, -4742 and -3125 strongly up-regulated and miR-1275, -3617 and -500b down-regulated. Logistic regression analysis revealed that measurement of three miRNAs (miR-423-5p, miR-23a-3p and miR-195-5p) could identify early-stage COVID-19 with 99.9% accuracy. As patients recovered from disease, the three-miRNA plasma signature returned to that of the healthy controls. The miRNA signature was shown to be robust in the ferret model of COVID-19 and could distinguish SARS-CoV-2 infection from seasonal influenza A infection. These findings suggest that miRNA profiling may be adopted to improve COVID-19 detection and patient management.

## Results

### Host miRNAs are altered in response to SARS-CoV-2 infection

Plasma samples were obtained from ten COVID-19 patients and ten age- and gender-matched healthy controls (Table 1). Longitudinal samples were available for some COVID-19 patients, categorized by visit (V), with V1 representing the plasma sample first taken following hospital admission (Table 2). Plasma samples were first obtained from COVID-19 patients 2–15 days (average 8 days) post symptomatic disease onset. Small RNA deep sequencing resulted in 23–50 million (average 34 million) raw reads per sample, which have been submitted to the NCBI short read archive (SRA). Reads were trimmed of adaptors and filtered on length and quality, resulting in a loss of 29–74% (average 56%) of raw reads, leaving 8–35 million (average 15.2 million) reads per sample for further analysis (S1 Fig). The majority of sequences were deemed high quality by FASTQC.

**Table 1:** Participant details.

	HEALTHY	COVID-19	P-value
**Participants, N**	10	10	-
**Female, %**	60	60	ns^#^
**Age, years** **(mean ± SD)**	53 ± 17.6	53.5 ± 17.2	ns^##^
**Required oxygen therapy, N (%)**	-	4 (40%)	-

ns = non-significant ^#^ Chi-Square test ^##^ Normality test and two-sided t-test.

**Table 2:** COVID-19 patient visit details.

COVID-19 Sample Visits	V1	V2	V3	V4
Days post symptom onset (mean ± SD)	8 ± 4	13 ± 6	38 ± 1	54 ± 18
Patient 1	X		X	
Patient 2	X	X		
Patient 3	X	X	X	
Patient 4	X			
Patient 5	X	X	X	
Patient 6	X	X	X	X
Patient 7	X^^^	X^^^		
Patient 8	X	X		
Patient 9				X
Patient 10				X

^^^Due to technical issues, these samples were not sequenced. However, all V1 samples were used for qRT-PCR validation.

MiRDeep2 was used to identify all known miRNA transcripts amongst the 29 samples and read counts were determined for each mature miRNA transcript. Total counts included all reads that mapped to a locus (as opposed to reads matching the canonical/consensus sequence only). A total of 985 different mature miRNA transcripts were detected, corresponding to 756 different precursors (5p and 3p miRNAs were counted separately). We did not observe a significant difference in the total number of miRNAs identified in infected versus uninfected patients. The most abundant miRNA in the plasma dataset was miR-16-5p, followed by miR-223-3p, let-7b-5p and miR-146a-5p.

**Fig 1:**
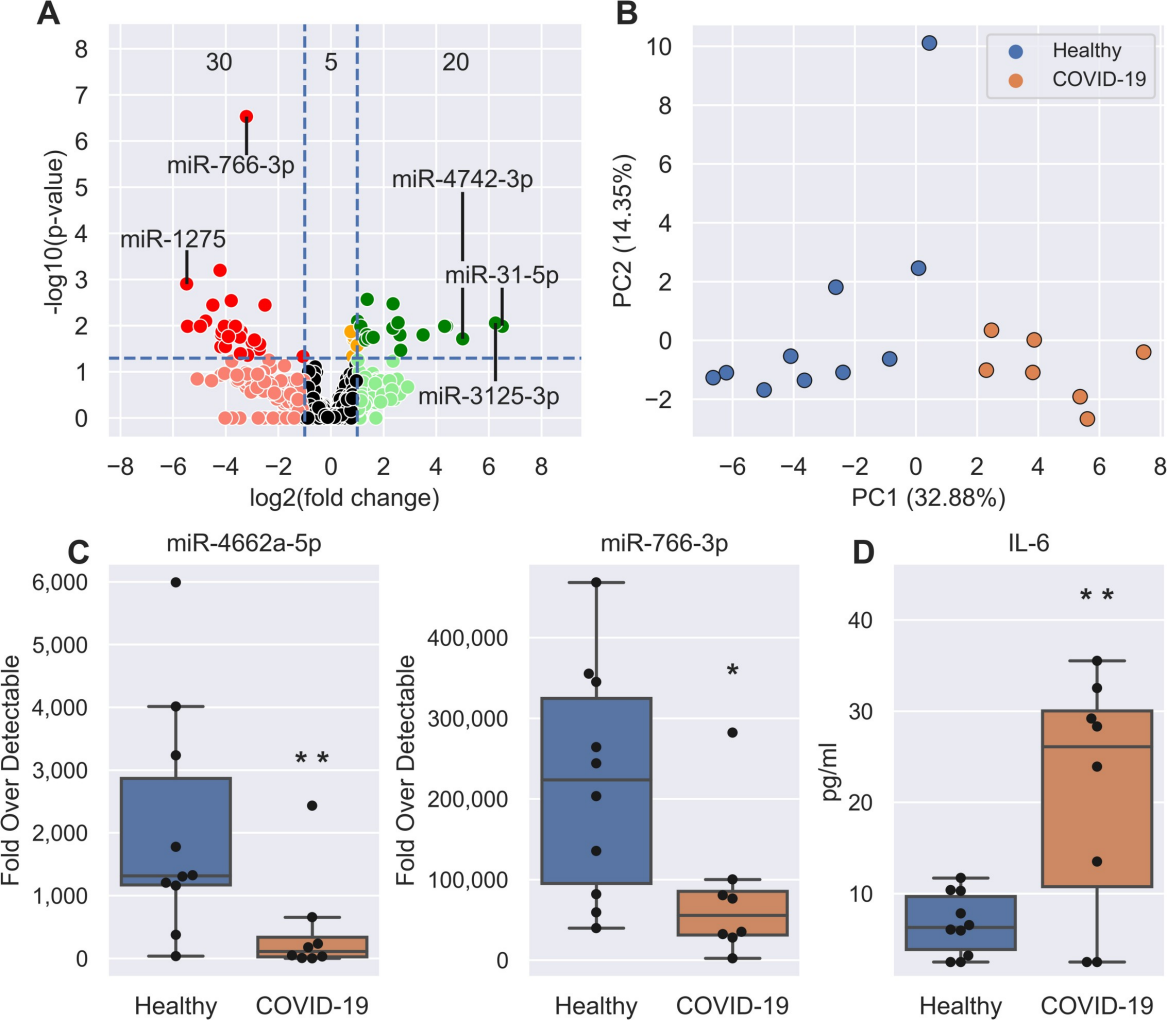
SARS-CoV-2 induces circulating miRNA and cytokine changes. **A,** Volcano plot showing the increased (green) and decreased (red) DE miRNAs in V1 COVID-19 patients when compared to healthy controls. Horizontal dotted line is the p-value cut-off (False Discovery Rate, FDR<0.05) and the vertical lines are the fold change cut-off (>2 FC). Orange miRNAs are statistically significant but are not >2 FC. The number of statistically significant miRNAs (adjusted P-value <0.05) in each section are shown: <-1 Log2 FC (30 miRNAs), between –1 and 1 Log2 FC (5 miRNAs), and >1 Log2 FC (20 miRNAs). The most up-regulated, down-regulated, and statistically significant miRNAs have been labelled. **B,** PCA plot showing the separation of healthy (blue, n = 10) and COVID-19 V1 (orange, n = 7) samples using the 55 DE miRNAs. **c-d,** Boxplots of (C) select qRT-PCR validated miRNAs and (D) IL-6 expression in healthy (blue, n = 10) and COVID-19 V1 (orange, n = 8) samples. Boxes are the 25^th^ - 75^th^ percentile, line is the median, and whiskers are 1.5x IQR. * p-value < 0.05, ** p-value < 0.01.

We sought to identify miRNAs with significantly altered expression levels between healthy control (n = 10) and COVID-19 V1 (n = 7) samples. This dataset consisted of 50 miRNAs, of which 20 were up-regulated (elevated in infected patients) and 30 were down-regulated (Fig 1A and S2 Table). An additional 5 miRNAs were significantly DE in COVID-19 patients with log_2_FC values <1. The most highly up-regulated candidates in COVID-19 patients were miR-31-5p (associated with inflammatory disorders [18–20]) (Fig 1A), miR-3125 and miR-4742-3p, while the most down-regulated were miR-1275 (Fig 1A), miR-3617-5p and miR-500b-3p. The most statistically significant change was seen in miR-766-3p (Fig 1A), a known anti-inflammatory miRNA[21]. Unsupervised analysis of variance using principal components analysis (PCA) involving the 55 DE miRNAs showed tight clustering of patient groups (Fig 1B). qRT-PCR was employed to validate select DE miRNA expression (Fig 1C). Quantitation of circulating cytokines highlighted a significant increase in IL-6 (Fig 1D) during acute COVID-19 illness, supporting previous studies [4]. Other patient cytokine data is shown in S3 Table.

### A three-miRNA signature accurately predicts COVID-19

**Fig 2:**
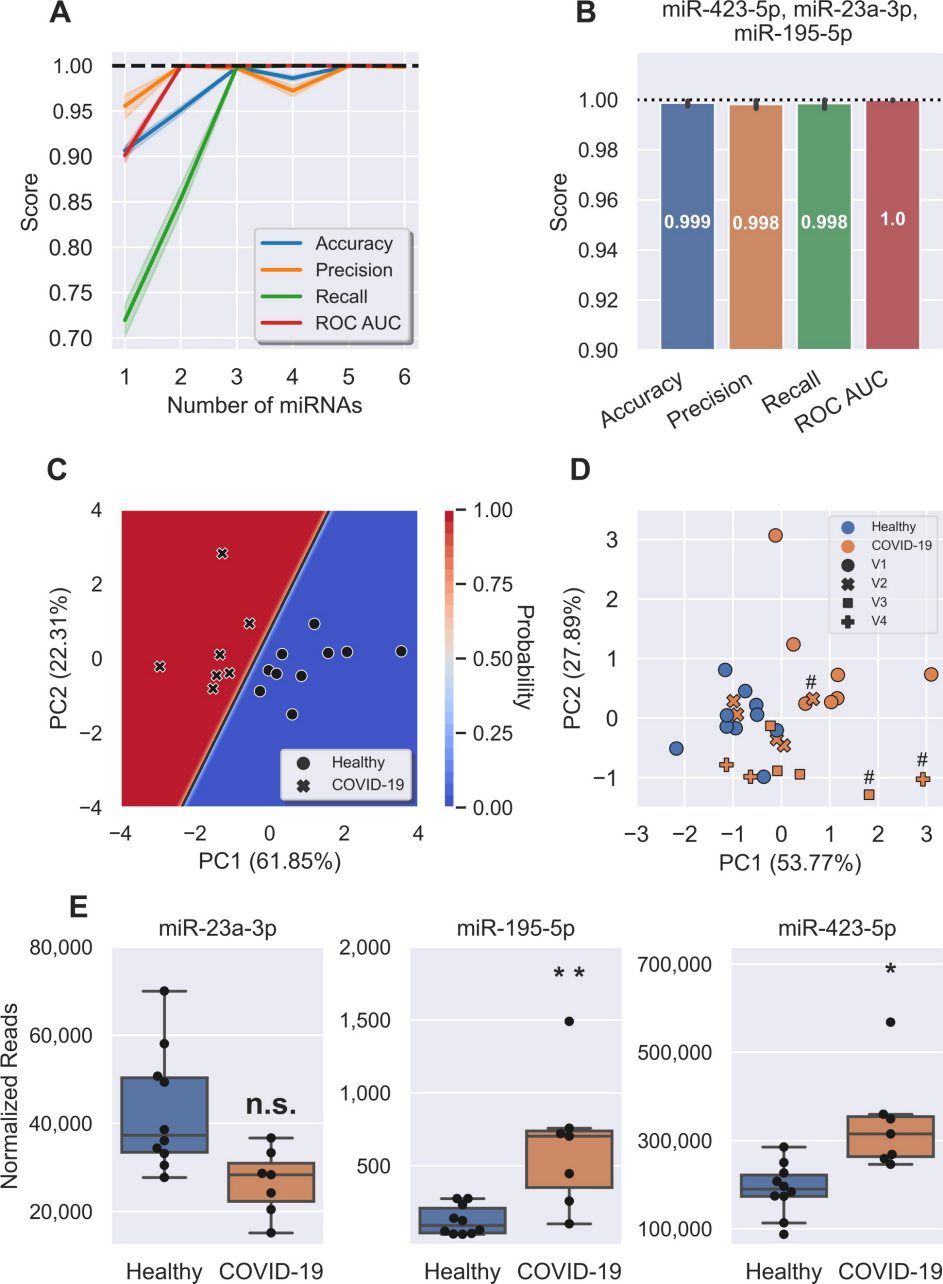
A three miRNA signature classifies COVID with 99.9% accuracy. **A,** Feature (miRNA) selection lineplot showing the impact of increasing numbers of miRNAs on the performance of a logistic regression model. MicroRNAs were selected using recursive feature elimination to identify the most important miRNAs. Each combination of miRNAs was randomly assessed 1,000 times. Shaded areas are the 95% CI, and the dotted line is a perfect (100%) score. **B,** Barplot showing the average score of the three-miRNA signature in predicting healthy controls and COVID-19 patients. Error bars are the 95% CI after 1,000 random iterative assessments. **C,** Decision boundary graph showing the logistic regression decision point (solid black line) and the probability a person is infected with SARS-CoV-2 (blue to red shading). Datapoints are healthy (circles, n = 10) and COVID-19 V1 (crosses, n = 7) samples. **D,** PCA plot based on the three miRNA signature showing all healthy (blue, n = 10) and COVID-19 (orange, n = 19) samples. Subsequent V2 (crosses, n = 5), V3 (squares, n = 4), and V4 (plus signs, n = 3) samples cluster with the healthy controls, apart from those denoted with a hash (#)–these all came from one participant that was treated in ICU and had not recovered at any visit. **E,** Boxplots of each of the signature miRNAs in healthy (blue, n = 10) and COVID-19 V1 (orange, n = 7) samples. Boxes are the 25^th^ - 75^th^ percentile, line is the median, and whiskers are 1.5x IQR. * FDR adjusted p-value < 0.05, ** FDR adjusted p-value < 0.01. n.s. non-significant.

Technologies most commonly utilized for COVID-19 diagnosis are virus-targeting molecular assays or serology, both of which can be associated with relatively high false-positive rates [22,23]. We therefore investigated whether, during the early stages of COVID-19, infected patients displayed a miRNA profile that could independently identify SARS-CoV-2 infection. A supervised machine learning method was implemented for the identification of the most predictive miRNAs and refined to identify the minimum targets necessary for accurate prediction and classification between healthy control and COVID-19 (V1) samples. A logistic regression model was implemented that randomly split the data into discovery and validation sets, trained and tested the model, which was repeated 1,000 times to determine reproducibility. The most predictive miRNAs were selected using recursive feature elimination (Fig 2A). Measuring a single miRNA (miR-195-5p) identified COVID-19 (V1) cases with ~90% accuracy, 95% precision, and 72% recall with a receiver operating characteristic area under the curve (ROC AUC) of 0.9. Measuring three miRNA targets (miR-423-5p, miR-23a-3p and miR-195-5p) in combination gave a model with 99.9% accuracy, 99.8% precision and 99.9% recall, with a ROC AUC of 1.0 (Fig 2B). Interestingly, the biomarker was comprised of two miRNAs DE in COVID-19 patients (miR-423-5p and miR-195-5p, both upregulated) and miR-23a-3p, which was not DE (Fig 2E). Increasing candidates within the biomarker signature to more than three miRNAs did not improve test performance.

A decision boundary graph showed clear distinctions between healthy and infected patients based on these three miRNAs (Fig 2C). The decision boundary graph also clearly shows that each sample’s grouping was predicted with a high degree of confidence (0% probability of healthy samples being identified as infected with SARS-CoV-2, and 100% probability of COVID-19 samples being detected as infected). The probability of a sample being infected with SARS-CoV-2 is determined by its distance from the decision boundary. The absence of points close to the boundary supports the high predictive accuracy of this miRNA signature. Interestingly, samples taken at successive timepoints (V2, V3 and V4) cluster with the healthy controls, indicating a return to normal baseline and suggesting that the three-miRNA signature is associated with the early stages of COVID-19 (Fig 2D). The obvious exception to this is one sample from V2, V3, and V4 that clusters closer to the V1 COVID-19 samples (indicated by # in Fig 2D); these samples all came from the same participant who was treated in the intensive care unit (ICU) and had not recovered from COVID-19 at any of the timepoints sampled. To further support the hypothesis that this signature detects early symptomatic COVID-19, we tested the model on the later time points (V2,3 and 4) saw the accuracy reduced to 16.6% (S2 Fig).

### Circulating miRNAs differ based on disease severity

Pro-inflammatory cytokines and chemokines (IL-6, IL-8, TNF-α, IL-1β) are differentially expressed in COVID-19 patients according to severity [5]. We therefore investigated prospective differences between miRNA profiles in moderate and severe COVID-19 cases. In this analysis the need for infected patients to receive supplemental oxygen (O_2_) or intubation was used as a proxy marker for severe disease, a metric previously used to categorise COVID-19 severity [4,5]. COVID-19 V1 patient samples were split into two groups (COVID-19 and COVID-19 + O_2_) based on the need for supplemental oxygen or intubation and then compared to the healthy controls. Analysis revealed that COVID-19 patients requiring oxygenation had fewer DE miRNAs in circulation (15 vs 42) compared to patients not requiring oxygenation (S3A Fig and S4 Table). Interestingly, four miRNAs (let-7e-5p, miR-651-5p, miR-766-3p, and miR-4433b-5p) were differentially expressed in both groups, suggesting that these molecules might be potential candidates for stratifying patients based on severity. Indeed, the healthy, COVID-19 and COVID-19 + O_2_ groups clustered based on the expression of these four miRNAs (S3 Fig). It is important to note the small number of samples involved in this analysis and that this finding requires validation in larger patient cohorts.

### The human COVID-19 signature also predicts ferret SARS-CoV-2 infection and differentiates it from influenza infection

**Fig 3:**
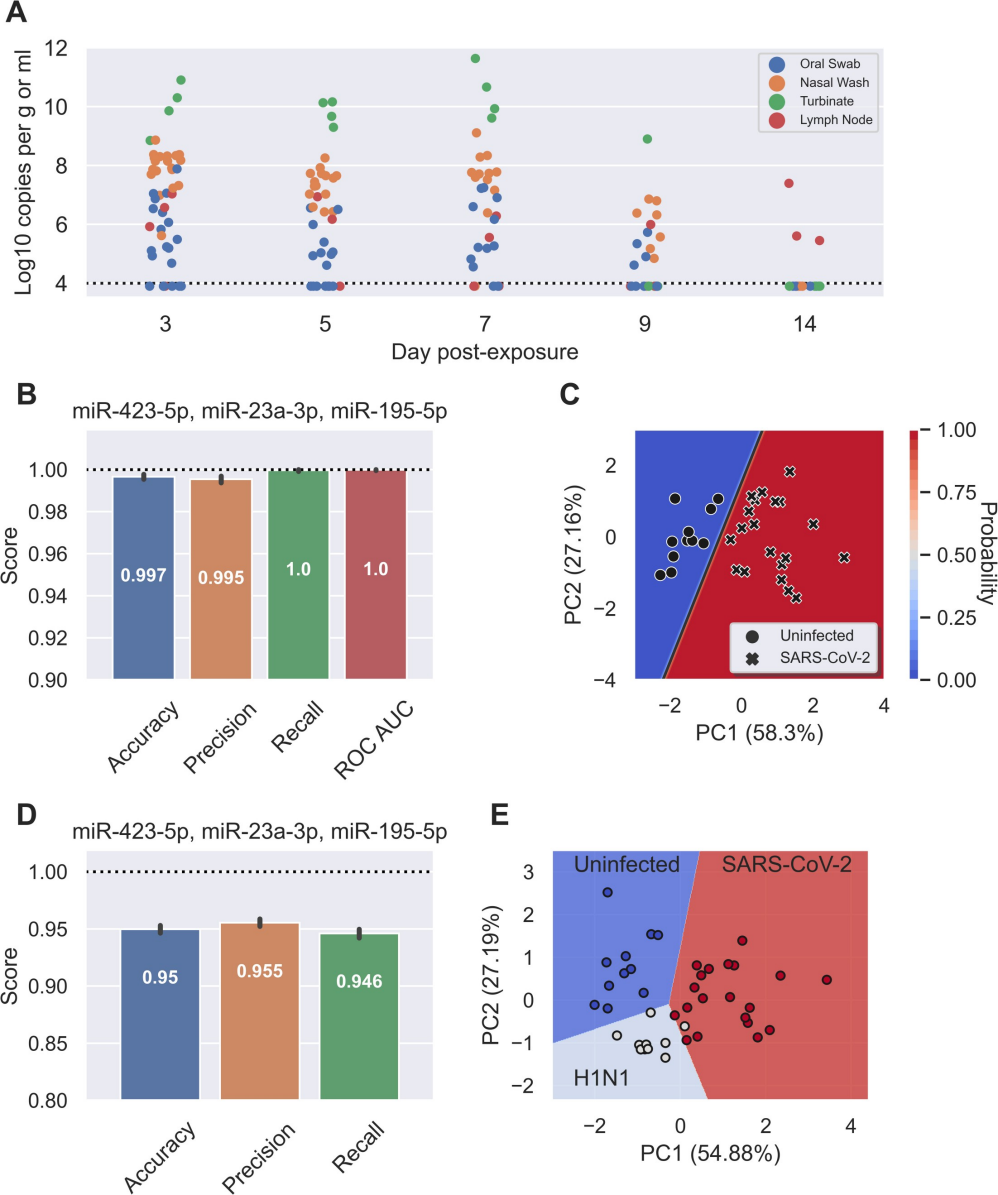
Human miRNA signature accurately identifies influenza and SARS-CoV-2 infection in a ferret model. **A,** Detection of SARS-CoV-2 viral genomic RNA in the retroperitoneal lymph node (blue), nasal wash (orange), oral swab (green), and turbinate tissue (red) of infected ferrets (n = 20, swab and wash samples taken from every ferret at each time point, tissue samples were analysed from the 4 euthanized ferrets at each time point). Data is presented as log10 copies per g of tissue or ml of sample. **B,** Final metrics of the trained logistic regression model to identify uninfected or SARS-CoV-2 infected ferrets. Dotted line is a perfect score (or 100%). Error bars are 95% CI for 1,000 random assessments. **C,** Decision boundary graph showing the logistic regression decision point (solid black line) and the probability a sample is infected with SARS-CoV-2 (blue to red shading). Datapoints are uninfected (circles, n = 11) and SARS-CoV-2 infected (crosses, n = 20) ferrets. **D,** Final metrics of the trained linear support vector classifier model to identify uninfected, influenza A (H1N1) virus, or SARS-CoV-2 infected ferrets. Dotted line is a perfect score (or 100%). Error bars are 95% CI for 1,000 random assessments. As ROC AUC is a measure of binary classification (two groups) it is omitted here. **E,** Decision boundary graph showing the linear support vector classifier decision points and predicted groups: uninfected (blue, n = 11), influenza A (H1N1) virus infected (light blue, n = 11) or SARS-CoV-2 infected (red, n = 20) ferrets.

Finally, we investigated whether the biomarker of early-stage COVID-19 was robust in an animal model and could distinguish between different viral respiratory infections. To address this, infection studies were performed in domestic ferrets (*Mustla putorius furo*), a well-established model for human respiratory viruses, including SARS-CoV-2 and influenza virus[24]. Twenty adult ferrets were exposed to SARS-CoV-2 via the intranasal route and monitored for clinical signs, with four ferrets euthanized at 3, 5, 7, 9, and 14 days post-exposure (d.p.e.). The establishment of infection was confirmed by performing qRT-PCR for viral genomic RNA on tissues and swabs (Fig 3A). High viral load was detected in nasal wash samples from day 3, which declined over time and was negative in all ferrets by 14 d.p.e. Eleven ferrets were infected with influenza A(H1N1) virus via the intranasal route, with animals euthanized at days 1, 2, 3, 5, 6 and 7 d.p.e. Influenza virology data in tissue and swab samples is shown in S4 Fig. Viral load was detected in nasal wash samples from 1 to 7 d.p.e..

Small RNA from serum samples were profiled for miRNAs using the same methodology as the patient samples. Sera from 12 uninfected ferrets were included as controls. In the ferret model, the previously identified biomarker signature (miR-423-5p, miR-23a-3p and miR-195-5p) could independently distinguish uninfected ferrets from COVID-19 infected ferrets with 99.7% accuracy, 99.5% precision, 100% recall, and a ROC AUC of 1.0 (Fig 3B). As with the human plasma samples, the decision boundary graph displayed high confidence in the predicted groupings (Fig 3C). Intriguingly, the miRNA biomarker still identified SARS-CoV-2 infection at 14 d.p.e., by which time ferrets were SARS-CoV-2 negative by nasal wash qRT-PCR, but with virus replication observed in the retroperitoneal lymph node tissue of 3 out of 4 ferrets (Fig 3A). In addition, the biomarker could distinguish SARS-CoV-2 infection from influenza infection and healthy control ferrets with 95% accuracy, 95.5% precision and 94.6% recall (Fig 3D). The decision boundary graph comparing predicted grouping and true grouping is shown in Fig 3E.

## Discussion

Here we present an unbiased profiling study of the circulating miRNAs in COVID-19 patients. In plasma samples obtained soon after the onset of disease symptoms (V1), a total of 55 miRNAs were DE, with several miRNAs more than 50-fold up-regulated (miR-31-5p, miR-3125, miR-4742-3p) or down-regulated (miR-1275, miR-3617-5p, miR-500b-3p) compared to basal miRNA expression levels in healthy donors. A response involving three miRNAs (miR-423-5p, miR-23a-3p and miR-195-5p) was consistently observed in COVID-19 patients at V1 and could independently classify SARS-CoV-2 infection with >99% accuracy. Ferret infection trials showed that this signature response was robust across species and was still valid during timepoints where SARS-CoV-2 replication was observed in internal organs but not in nasal wash samples. The biomarker was less predictive of SARS-CoV-2 infection in V2, V3 and V4 patient samples, suggesting this response is associated with early-stage COVID-19.

This signature was not determined based on FC differences in miRNA expression between infected and control groups, as miR-423-5p and miR-195-5p were relatively mildly up-regulated COVID-19 patients (log_2_FC 2.35 and 0.86, respectively), while miR-23-3p was non-significantly down-regulated (log_2_FC -0.64, adjusted p-value = 0.103). We hypothesize that a biomarker consisting of multiple miRNAs is more robust than one based on absolute or relative levels of a single miRNA. This multivariate approach, coupled with advanced machine learning analysis, can highlight a biomarker pattern that may not be identified via traditional DE analysis. In support of this, we note that the three-miRNA signature was robust in humans and ferrets, despite a relatively poor overlap in DE miRNAs observed in human and ferret COVID-19 samples (S5 Table). While miR-423-5p, miR-23a-3p and miR-195-5p measured in combination have not been defined previously as a biomarker for a specific disease, increased expression of circulating miR-423-5p is observed during heart failure [25] and pulmonary tuberculosis [26]. Increases in circulating miR-195-5p are associated with osteosarcoma [27], autism [28] and gestational diabetes mellitus [29]. Interestingly, increased plasma expression of miR-195-5p is also observed during HIV-1 infection, with miR-195-5p forming part of a four-miRNA signature that can identify HIV-1 infection with high confidence [30].

While host responses to infection are known to be critical in differential outcomes of SARS-CoV-2 infection, the role of miRNAs in COVID-19 pathogenesis is poorly understood. We observed that miR-31-5p was the most strongly up-regulated miRNA in COVID-19 patients, which may be related to its role in modulating inflammation. Transcription of miR-31-5p in endothelial cells is induced by TNF-α and triggers a negative feedback loop involving E-selectin to control inflammatory signalling [31]. MicroRNA-31-5p is also upregulated in inflamed ulcerative colitis mucosa [32], where it downregulates expression of the IL-13 receptor α-1 (*IL13RA1*) gene, which diminishes expression of signal transducer and activator of transcription 6 (*STAT6*), suppressor of cytokine signalling 1 (*SOCS1*) and eotaxin-3 (*CCL26)* expression. These findings raise the possibility that miR-31-5p is induced in response to acute stressors such as SARS-CoV-2 in order to curtail an excessive inflammatory response. Interestingly, miR-27a-5p (also up-regulated in V1 COVID-19 samples), is elevated in animal models of enterocolitis [33]. The up-regulation of miR-31-5p and miR-27a-5p in COVID-19 patients may reflect SARS-CoV-2 mediated gastrointestinal tract infection or inflammation [34]. Furthermore, the most statistically significant down-regulated miRNA was miR-766-3p, a previously identified anti-inflammatory miRNA. This miRNA was shown to reduce the expression of IL-6 in TNF-α stimulated MH7a cells [21] and so its reduction may be partially responsible for the characteristic IL-6 increase seen in COVID-19 patients [4]. In addition to miR-31-5p, miR-27a-5p, and miR-766-3p, we observed several miRNAs DE in COVID-19 patients that are poorly characterized from a functional perspective. Many miRNAs upregulated (miR-3125, miR-4742-3p, miR-2116-3p) or down-regulated (miR-3617-5p, miR-500b-3p, miR-3684) in COVID-19 patients have not been functionally characterized or previously observed in studies of miRNA responses to viral infection.

Current COVID-19 molecular tests target viral RNA for detection. Unfortunately, even the most advanced current molecular diagnostic tests (i.e. PCR or LAMP amplifying viral RNA) for SARS-CoV-2 require a relatively high viral load to accurately detect infection[35]. Thus, their sensitivity during the early pre-symptomatic phase of disease (incubation period), when the viral load is still low, is poor. Overall sensitivity of current PCR tests has been estimated to be as low as 30–70%[22,23], making it difficult to diagnose infections in many pre-symptomatic and some asymptomatic cases. Our study suggests that SARS-CoV-2 infection induces a miRNA response during the early stages of disease that involves three miRNAs (miR-423-5p, miR-23a-3p and miR-195-5p) that can independently identify COVID-19 cases and distinguish SARS-CoV-2 from influenza infections. Further studies involving larger patient groups, including pre-symptomatic, asymptomatic and mild (non-hospitalised) patients, in addition to different infections, are planned to assess whether this miRNA biomarker can improve COVID-19 detection rates. While the present study did not measure circulating miRNA profiles in patients infected with other respiratory viruses, published reports of circulating miRNAs DE in influenza A and influenza B patients [36] show clear differences to COVID-19 patients. These findings, in addition to data from ferret infection trials in the present study, indicate that miRNA profiling may be able to classify different infection types.

In summary, this study exemplifies how analysis of miRNA responses to SARS-CoV-2 infection presents novel avenues in the characterization of cellular factors aiding in COVID-19 pathogenesis. It also presents novel opportunities for treatment and diagnosis of viral diseases. Targeting of pro-inflammatory miRNAs could present novel therapeutic opportunities against COVD-19, while miRNA profiling may aid in the disease detection and surveillance.

## Method

### Ethics statement

Human experimental work was conducted according to the Australian National Health and Medical Research Council Code of Practice. The study was approved by the Alfred Hospital (#280–14) and The University of Melbourne (#2056761, #1443389 and #1955465) Human Research Ethics Committees. The analysis of miRNAs from patient samples was approved by the CSIRO Human Research Ethics Committee (proposal # 2020_19). Formal consent was not obtained from patients due to anonymity. All animal studies were approved by the CSIRO Australian Centre for Disease Preparedness Animal Ethics Committee (document 1990 for SARS-CoV-2, document #1568 for influenza A(H1N1)) and conducted following the Australian National Health and Medical Research Council Code of Practice for the Care and Use of Animals for Scientific Purposes guidelines for housing and care of laboratory animals. All work with live animals was approved by the Institutional Animal Ethics Committee in accordance with guidelines from the Australian Code for the Care and Use of Animals for Scientific Purposes (8th Edition) and compliant with the Victoria State Prevention of Cruelty to Animals Act 1986 and Part 5 of the Prevention of Cruelty to Animals Regulations 2019. The facility has assurance from the US Office of Laboratory Animal Welfare Assurance (Legacy Assurance ID A5399-01). All animals were acclimatized for at least 7 days prior to entering the study, given food and water ad libitum, and monitored daily. Environmental enrichment was also provided in the cages during the study.

### Patient cohort information

Plasma samples were collected from patients admitted to the Alfred Hospital (Melbourne, Australia) from February to April 2020. Plasma samples were also collected from patients defined as healthy controls (i.e. pre-exposure), who displayed no COVID-19 symptoms and returned negative COVID-19 PCR test prior to sample collection. Patient metadata is shown in S1 Table. The ethnicity of the cohort was not reported.

### Ferret infection trials

Twenty outbred ferrets (10 male and 10 female, approximately four months of age) were exposed to 4.64 × 10^4^ TCID_50_ of SARS-COV-2 (hCoV-19/Australia/VIC01/2020) [37] by the intranasal route. Prior to any manipulations, animals were immobilised with a mixture of ketamine HCl (5 mg/kg) and medetomidine (0.05 mg/kg); atimepazole was administered for reversal at a dose of 0.25 mg/kg. After virus exposure, animals were monitored for clinical signs of disease, and fever. They were randomly assigned to euthanasia on post-exposure days 3, 5, 7, 9 or 14, when clinical samples including nasal washes, serum and urine were collected together with multiple tissue specimens. Eleven ferrets (5 male and 6 female, aged 4–6 months) were exposed to 1 x 10^5^ TCID50 of influenza A (H1N1) (A/California/07/2009) virus as described [38]. At the terminal sampling point, ferrets were anaesthetised as described above and exsanguinated via cardiac puncture before humanely killed via injection of sodium pentobarbitone (up to 150 mg/kg) while under anaesthesia. After confirmation of death, a necropsy was performed and a panel of swab and tissues (including oral swab, nasal swab, turbinate and lymph node) collected without fixation for virological assessment. Tissues were weighed, before homogenisation in 1 mL of transport media (PBS + 0.1% BSA) using a FastPrep-24 (MP Biomedicals), of which 50 μL was transferred to 260 μL of MagMax buffer for viral RNA extraction.

### RNA isolation and heparinase treatment

For microRNAs, total RNA was isolated from 200 μL of human plasma and 150 μL of ferret serum using the miRNeasy micro kit (Qiagen) as per the manufacturer’s instructions with one modification: glycogen (10 μg, Sigma Aldrich, G1767) was added as a carrier to each sample after lysis with Qiazol. As the human plasma samples were originally obtained using sodium heparin vacutainers, the eluted RNA was treated with 1U heparinase I (Sigma Aldrich, H2519) at 25°C for 30 min to remove any remaining heparin. For viral RNA, RNA was extracted using the Mag-Max Viral RNA isolation kit (Thermo Fisher Scientific).

### Next-generation sequencing

Complementary DNA libraries were generated using the QIAseq miRNA Library Kit and QIAseq miRNA NGS 48 Index IL (Qiagen) as per the manufacturer’s protocol (handbook HB-2157-007 March 2020), with slight modifications: 5 μL of eluted RNA was used as the template and the libraries underwent 24 cycles of amplification. All libraries were analysed on the Bioanalyser 2100 using the High Sensitivity DNA Kit (Agilent) to ensure correct insert size and minimal adapter or primer carryover. Libraries were then sent to the Australian Genome Research Facility (AGRF) for 100 bp single end sequencing on the NovaSeq 6000 (Illumina). Due to technical issues, 1 V1 COVID samples could not be sequenced but was used in qRT-PCR validation.

### Data pre-processing and differential expression

Adapters were trimmed using [39] with a read length parameter (18–26 nucleotides). The remaining reads were examined using FastQC (www.bioinformatics.babraham.ac.uk/projects/fastqc/) to ensure high-quality data. miRDeep2 quantifier [40] was used to map and quantify reads against the latest miRBase human reference (version 22 [41]). Raw read counts were normalized and differential expression analysis was completed using the DESeq2 [42] package in R. An adjusted False Discovery Rate (FDR) p-value of <0.05 was used to identify differentially expressed miRNAs. DESeq2 was used to perform count-based differential expression (DE) testingusing a False Discovery Rate (FDR) adjusted p-value <0.05, log_2_ fold change (FC) >1 and baseMean >5.

### Machine learning

All machine learning analysis was conducted using the scikit-learn [43] module in python. Normalized reads were first examined for highly correlated miRNAs; any pairs with a Pearson R of >0.8 or <-0.8 had one member removed. Highly correlated features (miRNAs) can impact the performance of machine learning algorithms. Multicollinearity can cause skewed or misleading results, especially in models such as logistic regression. The remaining normalized miRNA counts were scaled using either a standard z-score transformation or a robust scaler (where the median is removed and the data is scaled according to the interquartile range). Feature selection was performed using recursive feature elimination (RFE) to identify the miRNAs that contributed the most to the classification model. For binary classification, a logistic regression model was used. For multiclass classification, a linear support vector classifier was used. Once the optimal number of features (miRNAs) was selected, the data was PCA transformed. Each model underwent hyperparameter tuning using GridSearchCV. To assess the performance of the classification model, the data was randomly split into 70% labelled training data and 30% unlabelled test data, and the predicted classes of the test data samples were compared to the true classes. This process was repeated 1,000 times to ensure confidence in the classification performance. The machine learning models were assessed on their accuracy (how many of the predictions were correct), precision (how many of the predicted positives were true positives), and recall (how many of the true positives were found by the model). The logistic regression model was also assessed using the receiver operating characteristic area under the curve (ROC AUC), which is a succinct metric to describe a binary classification model [12].

### qRT-PCR

MicroRNA cDNA was generating using the TaqMan Advanced miRNA cDNA Synthesis Kit (Applied Biosystems) with 2 μL of input RNA as per manufacturer’s instructions. qPCR was conducted using 1X TaqMan Advanced miRNA Assay, 1X TaqMan Fast Universal PCR Master Mix, no AmpErase UNG (Applied Biosystems) and 5 μl of 1:10 diluted cDNA product, using standard PCR cycling conditions (95°C for 20 sec, 40 cycles of 95°C for 1 sec, 60°C for 20 sec). Cycle threshold for all assays was set to 0.1. Data is presented as fold over detectable, as previously described [44], with a detectability cut off of C_T_ = 40. For detection of SARS-CoV-2 in human samples, qRT-PCR was performed using the Highplex/Corona virus Typing (8plex) Assaytype multiplex Tandem PCR (AusDiagnostics, Australia) according to manufacturer’s instructions. For ferret samples, The presence of SARS-CoV-2 RNA were evaluated by qRT-PCR targeting the SARS-CoV-2 E gene using an AgPath-ID One Step RT-PCR kit (Thermo Fisher Scientific) with the following primers and Taqman probe: CoV-E-fwd (5’-AGTACGAACTTATGTACTCATTCGTT-3’), CoV-E-R2 (5’- ATATTGCAGCAGTACGCACACA -3’) and CoV E Probe 5’-6-FAM-ACACTAGCCATCCTTACTGCGCTTCG-MGB-3’) [45].

### Cytokine analysis

Plasma was diluted 1:2 and cytokine abundance measured using the LEGENDplex Human Inflammation Panel 1 kit, as per manufacturer’s instructions (BioLegend).

### Statistics

Statistical analyses were completed using the SciPy v1.6.0 package [46]. All measurements were taken from distinct samples. Differences in qRT-PCR results were examined using a one-sided Mann-Whitney U test due to the non-parametric nature of the fold-over-detectable transformation. Normality was tested using a combination skew and kurtosis test (scipy.stats.normaltest()). Differences in IL-6 expression was assessed using a one-sided t-test. A p-value <0.05 was considered significant. All statistics and p-values can be found in S6 Table.

## Supporting information

S1 Fig Data quality control. After adaptor trimming, reads that fell outside the expected size range for miRNAs (18–26 nt) were filtered out, as were reads that failed to map to a miRNA precursor.
(TIFF)

S2 Fig Application of the V1 COVID-19 miRNA signature to subsequent time points. Decision boundary graph showing the logistic regression decision point (solid black line) and the probability a person is infected with SARS-CoV-2 (blue to red shading). Datapoints are COVID-19 patients at V2 (circles, n = 5), V3 (crosses, n = 4), and V4 (squares, n = 3).
(TIF)

S3 Fig Differential miRNA profiles based on COVID-19 severity. a, Venn diagram of COVID-19 (light blue) and COVID-19 + O2 (yellow) V1 DE miRNAs when compared to healthy controls. b, PCA plot based on the four common DE miRNAs. Healthy (blue, n = 10), COVID-19 (orange, n = 3) and COVID-19 + O2 (green, n = 4) V1 samples.
(TIF)

S4 Fig Detection of influenza (H1N1) genomic RNA in lung tissue (blue), nasal swab (orange), nasal wash (green) and serum (red) of infected ferrets (2–4 ferrets per time point). Data is presented as normalized CT on a reverse y-axis. Undetectable results are plotted as CT = 40.
(TIF)

S1 Table Additional patient metadata. (XLSX)

S2 Table Differentially expressed miRNAs in V1 COVID-19 patients. (XLSX)

S3 Table Human cytokine data. (XLSX)

S4 Table Differentially expressed miRNAs in COVID-19 patients with and without oxygen therapy compared to healthy controls. (XLSX)

S5 Table Differentially expressed miRNAs in ferrets infected with SARS-CoV-2 compared to uninfected ferrets. (XLSX)

S6 Table Other statistical comparisons. (XLSX)
