# Supplementary figures and images for: Altered microRNA expression in COVID-19 patients enables identification of SARS-CoV-2 infection
Source: PLoS Pathog. 2021 Jul 28;17(7):e1009759. doi: 10.1371/journal.ppat.1009759 (PMC8318295; doi:10.1371/journal.ppat.1009759)

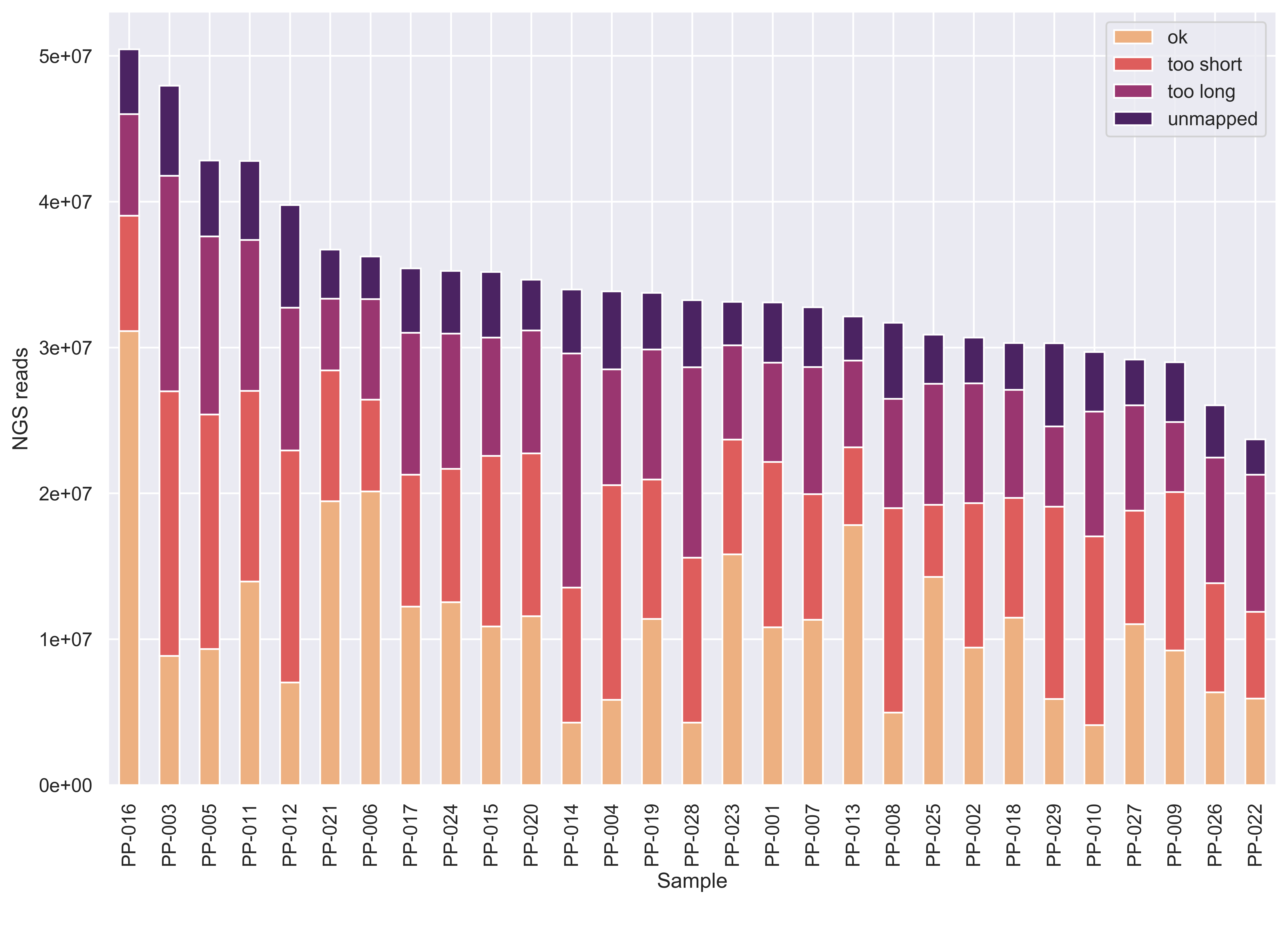

Supplement: S1 Fig — After adaptor trimming, reads that fell outside the expected size range for miRNAs (18–26 nt) were filtered out, as were reads that failed to map to a miRNA precursor. (TIFF) [file ppat.1009759.s001.tiff]

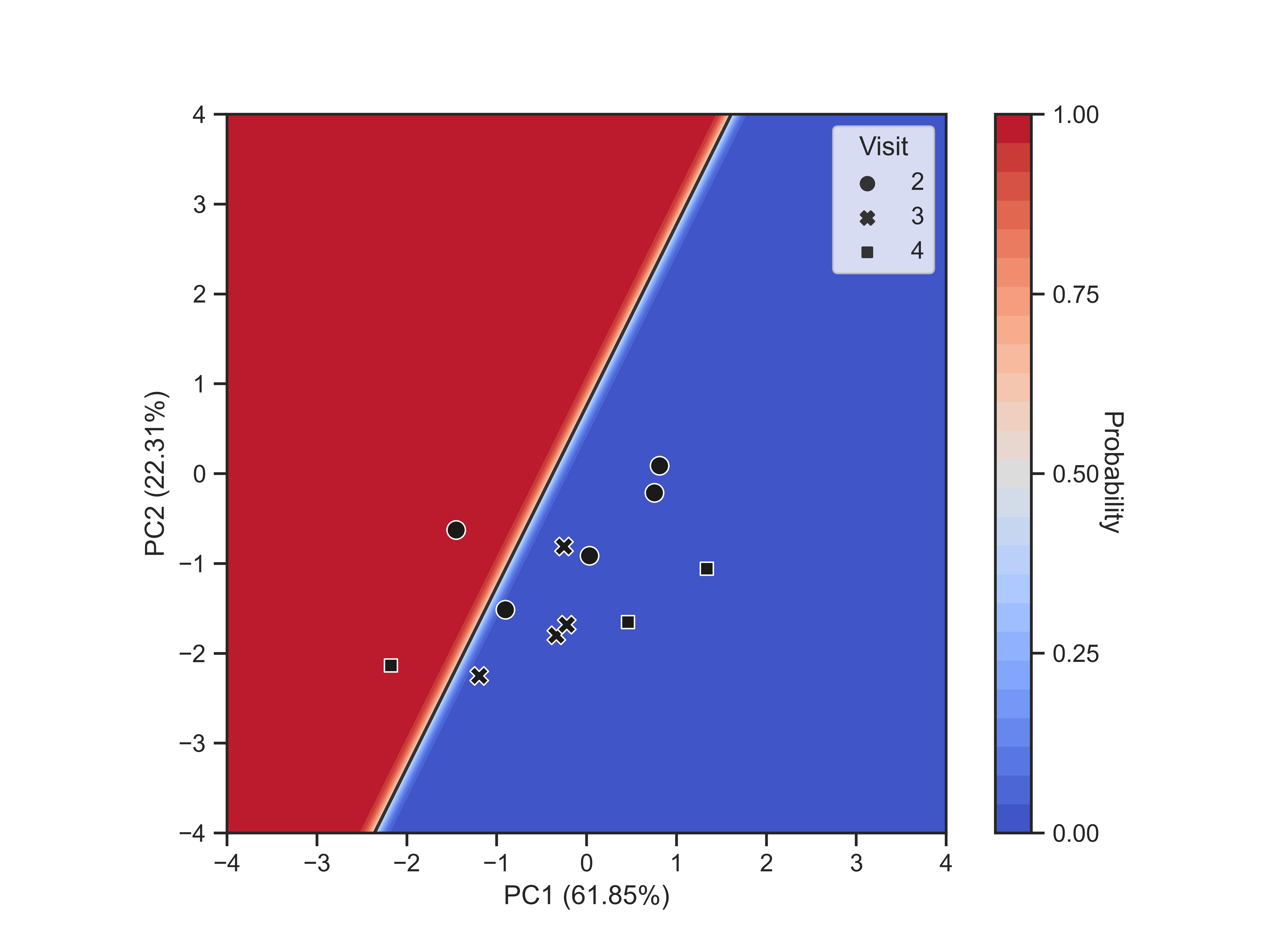

Supplement: S2 Fig — Decision boundary graph showing the logistic regression decision point (solid black line) and the probability a person is infected with SARS-CoV-2 (blue to red shading). Datapoints are COVID-19 patients at V2 (circles, n = 5), V3 (crosses, n = 4), and V4 (squares, n = 3). (TIF) [file ppat.1009759.s002.tif]

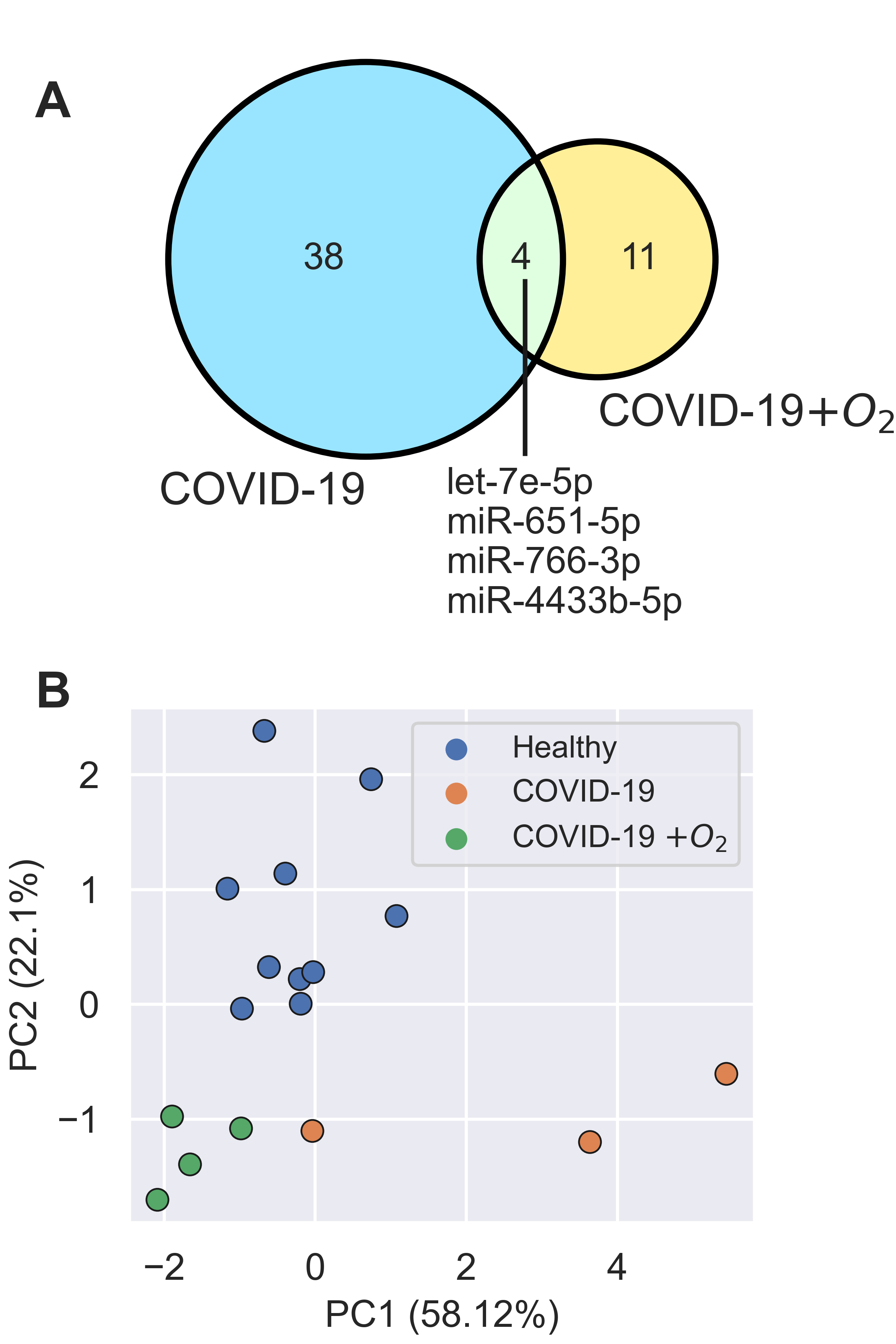

Supplement: S3 Fig — a, Venn diagram of COVID-19 (light blue) and COVID-19 + O2 (yellow) V1 DE miRNAs when compared to healthy controls. b, PCA plot based on the four common DE miRNAs. Healthy (blue, n = 10), COVID-19 (orange, n = 3) and COVID-19 + O2 (green, n = 4) V1 samples. (TIF) [file ppat.1009759.s003.tif]

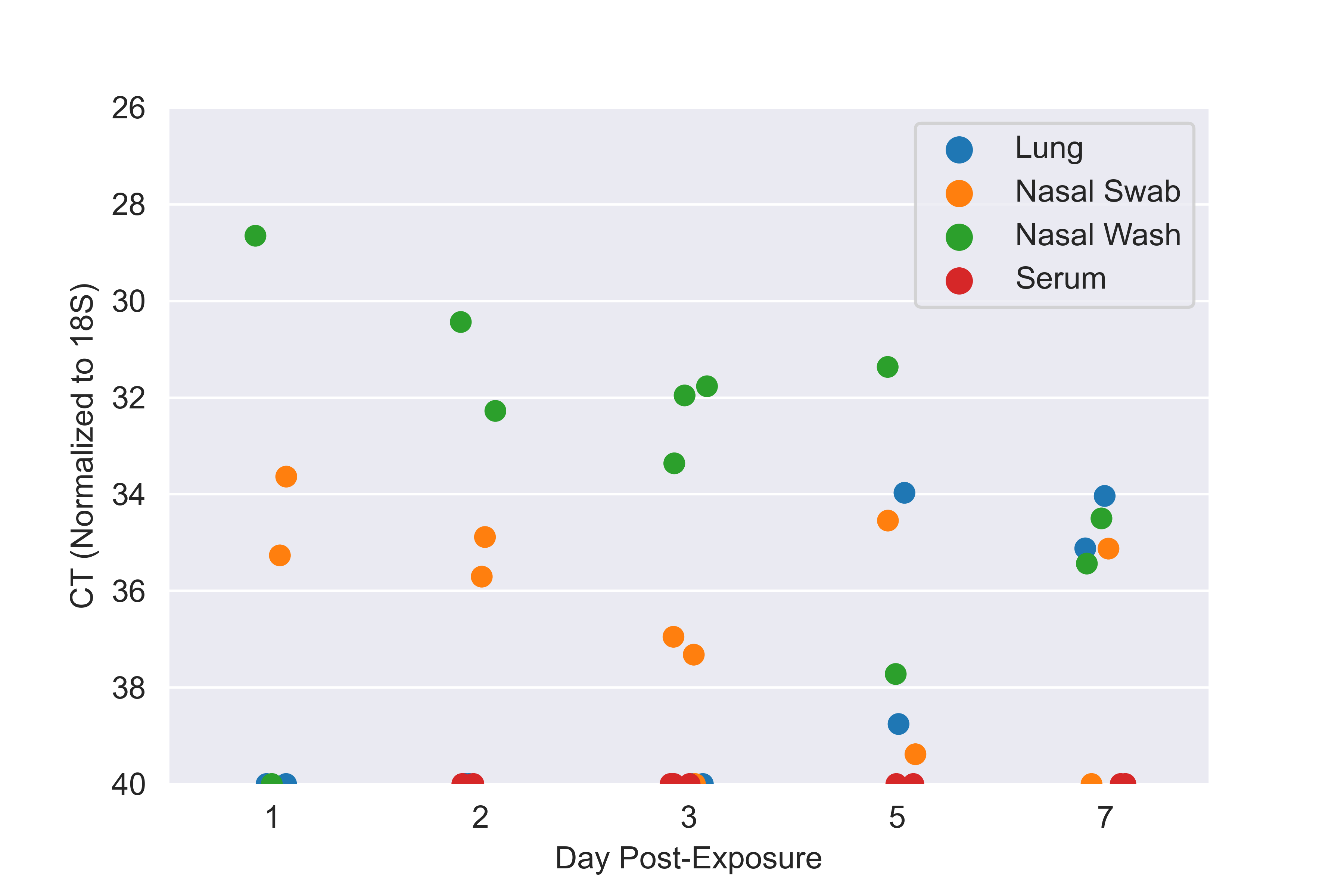

Supplement: S4 Fig — Detection of influenza (H1N1) genomic RNA in lung tissue (blue), nasal swab (orange), nasal wash (green) and serum (red) of infected ferrets (2–4 ferrets per time point). Data is presented as normalized CT on a reverse y-axis. Undetectable results are plotted as CT = 40. (TIF) [file ppat.1009759.s004.tif]
